# Developing a new score system for patients with PSA ranging from 4 to 20 ng/mL to improve the accuracy of PCa detection

**DOI:** 10.1186/s40064-016-3176-3

**Published:** 2016-09-05

**Authors:** Yuxiao Zheng, Yuan Huang, Gong Cheng, Cheng Zhang, Jie Wu, Chao Qin, Lixin Hua, Zengjun Wang

**Affiliations:** State Key Laboratory of Reproductive Medicine, Department of Urology, First Affiliated Hospital of Nanjing Medical University, 300, Guangzhou Road, Nanjing, 210029 Jiangsu Province China

**Keywords:** Prostate cancer, Score system, Diagnosis, Receiver operating characteristic curve, Sensitivity, Specificity

## Abstract

**Objective:**

To develop a new score system for patients with prostate specific antigen (PSA) ranging from 4 to 20 ng/mL to improve the accuracy of prostate cancer (PCa) detection, and to evaluate it with receiver operating characteristic curve.

**Methods:**

A total of 797 patients (208 with prostate cancer) with total PSA 4–20 ng/mL who had undergone transrectal ultrasound (TRUS)-guided 12 + 1-core prostate biopsy during Sept. 2009–Jan. 2013 were retrospectively evaluated in the study. Age, PSA, fPSA, PV, f/T, PSAD, DRE findings and ultrasound findings were considered as predictive factors and tested by logistic regression. Predictors with P < 0.05 were selected to develop a new score system.

**Results:**

Age, PSA, PV, f/T, DRE findings, and hypoechoic in ultrasound were selected in our new score system. The risk of PCa increased with the score. From 0 to 6, the risk was 2.0, 8.4, 13.9, 33.5, 63.8, 75.0 and 100.0 % respectively. Area under curve (AUC) of our new score system was 0.804, which was significantly higher than The Prostate Cancer Risk Calculator by Stichting Wetenschappelijk Onderzoek Prostaatkanker (SWOP) (0.720, P = 0.002).

**Conclusions:**

We developed a new simple score system for patients with PSA ranging from 4 to 20 ng/mL to improve the accuracy of PCa detection.

## Background

Prostate specific antigen (PSA) is widely used for the screening of prostate cancer in recent years. However, an increasing level of PSA can also represent the occurrence of benign prostate hyperplasia (BPH) and prostatitis, which questioned the specificity of PSA in predicting prostate cancer (Catalona et al. 1995). Although the region of PSA levels from 4 to 10 ng/mL is defined as a “gray zone” according to the traditional concepts, the prostate cancer detection rate of the patients with PSA levels of 10–20 ng/mL is not superior to the patients whose PSA levels in the “gray zone” conspicuously. The incidence rate was 25 % or less in the region of PSA from 4 to 20 ng/mL (Tang et al. 2013; Chavan et al. 2009). The rate of the patients with the PSA level in range of 4–20 ng/mL in the initial examination in clinic has been increase since the examination of PSA level was a routine physical examination. Clearly, there is an urgent need to develop a new system for improving the detection rate and reducing unnecessary prostate biopsies in the region of PSA levels from 4 to 20 ng/mL.

## Methods

### Ethics statement

This study was approved by the institutional review board of the First Affiliated Hospital of Nanjing Medical University. Written informed consent was obtained from all patients with regard to the storage of their information for the purpose of research. All research procedures were conducted in accordance with the Declaration of Helsinki.

### Characteristics and methods

Our study included 797 patients, who had an elevated PSA level ranging from 4 to 20 ng/mL and had undergone a transrectal ultrasound (TRUS)-guided prostate biopsy from September 2009 to January 2013 at the First Affiliated Hospital of Nanjing Medical University, China. All the patients envolved in our study are Chinese men. Detail patient information, including age, PSA, free PSA (fPSA), digital rectal examination (DRE) findings and other clinical information were recorded before prostate biopsy. The information of patients’ PSA levels were provided by laboratory of the First Affiliated Hospital of Nanjing Medical University, they used a single validated assay to assess PSA levels. An ellipsoid formula (PI/6 × lateral × anteroposterior × superoinferior diameters) was used to calculate the prostate volume (PV) of every patient in this study by means of ultrasonoscopy, and detailed observation were carried out regarding microcalcifications and hypoechoic lesions. PSA density (PSAD) was defined as the ratio of PSA to PV. The free/total PSA ratio (f/T) was calculated by dividing the level of fPSA present by the total level of PSA.

We used Philips Envisor C Ultrasound Machine to localized the position and used Bard Maxcore Disposable Biopsy Instrument MC1820 to collected the tissue of prostate. The TRUS-guided prostate biopsy from September 2009 to January 2013 were performed by experienced urologists in the First Affiliated Hospital of Nanjing Medical University. Routine examination including blood, liver and kidney function and coagulation was taken by the patients before the prostate biopsy.

### Statistical analysis

Statistical analysis was performed using SPSS 18.0 software. Differences between data of the patients were analyzed using the *t* test for continuous variables and the Chi square test for categorical variables. We used multiple logistic regression analysis with a backward elimination selection procedure to select potential predictors. One score was given when the positive factors more than the median score. 100 patients from a total of 797 patients with total PSA 4–20 ng/mL who had undergone TRUS-guided 12 + 1-core prostate biopsy during Sept. 2009–Jan. 2013 the First Affiliated Hospital of Nanjing Medical University were randomly selected with random number table for assessment of effectiveness of the new score system and The Prostate Cancer Risk Calculator by Stichting Wetenschappelijk Onderzoek Prostaatkanker (SWOP), which is an initiative of the Department of Urology of the Erasmus MC, the University and Medical Centre of Rotterdam, in The Netherlands. Erasmus MC is one of eight European centers taking part in the world’s largest study of screening for prostate cancer, the European Randomized Study of Screening for Prostate Cancer (ERSPC). We used a receiver-operating characteristic (ROC) curve to assess the effectiveness of the new score system and The Prostate Cancer Risk Calculator by SWOP Finally, Z-test was used to compare the difference of the area under the curve. We considered statistical significant when P value <0.05.

## Results

In this study, 208 (27 %) of 797 patients had positive biopsy results. The characteristic details of the patients in the study cohort are indicated in Table 1. The mean ages of patients in the PCa group and the non-PCa group were 70.6 ± 6.8 and 66.9 ± 8.7 years; P < 0.01. Moreover, other statistical differences including PSA (P < 0.001), fPSA (P < 0.001), PSAD (P < 0.001), f/TPSA (P < 0.001) DRE findings (P < 0.001) and hypoechoic under transrectal ultrasound findings (P < 0.001) were found between the PCa group and non-PCa group. However, there is no significant statistical difference in microcalcifications (P = 0.262). Univariate analysis indicated that age, PSA, fPSA, f/T, PV, PSAD, abnormal DRE and the rate of ultrasonic hypoechoic mass in patients with a positive initial prostate biopsy were different from patients with an initial negative biopsy. Age, PSA, fPSA, t/T, PV, PSAD, DRE findings and ultrasonic hypoechoic mass were included in logistic analysis on multivariate analysis. The categorical variables were valuated before analysis. We valuated abnormal and no significant abnormal DRE findings as 1 and 0 score; valuated hypo echoic mass and no significant hypoechoic mass in TURS as 1 and 0 score; valuated positive and negative prostate biopsy finding as 1 and 0 score. All the other independent variable were included in the logistic analysis as continuous variables. After a backward elimination selection procedure, six predictors indicated significant differences (Age, PSA, t/T, PV, DRE findings and ultrasonic hypoechoic mass in TURS) suggested that these parameters were potential predictors for initial prostate biopsy (Table 2). According to the result of the logistic analysis, the new system model was created including the significantly potential predictors such as age, PSA, f/T, PV, DRE findings and hypo echoic mass in TURS. The section point was defined as the overall median of the predictors. Positive factors such as age > 69, PSA > 9.3 was valuated as 1 score; while negative factors such as PV < 37.1, f/T < 0.14 was valuated 1 score. Additionally, abnormal DRE findings and hypo echoic mass in TURS was also valuated as 1 score. The positive rate of new score system is shown in Table 3.

**Table 1:** Characteristics of the patient cohort in the first stage of the study

Variables	Non-PCa Group	(%)	PCa Group	(%)	Overall median	P
Number of patients	589		208			
Age	66.9 ± 8.3		70.6 ± 6.8		69	<0.001
PSA	9.8 ± 4.0		11.2 ± 4.2		9.3	<0.001
fPSA	1.6 ± 1.2		1.3 ± 0.8		1.3	0.003
PV	46.2 ± 23.6		33.8 ± 13.7		37.1	<0.001
PSAD	0.25 ± 0.17		0.38 ± 0.20		0.24	<0.001
f/T	0.17 ± 0.11		0.12 ± 0.06		0.14	<0.001
DRE findings						<0.001
Neg	545	78.6	148	21.4		
Pos	44	32.3	60	57.7		
Hypoechoic^a^						<0.001
Neg	468	78.8	126	21.2		
Pos	121	59.6	82	40.4		
Microcalcification^a^						0.262
Neg	415	74.6	141	25.4		
Pos	174	72.2	67	27.8		

Values are mean ± SD and number

Student’s *t* test for age, PSA, fPSA, PV, PSAD and f/t distributions between Pca and Non-Pca groups

Two-sided χ^2^-test or Fish’s exact test for DRE findings, hypoechoic, and microcalcification between Pca and Non-Pca groups

*DRE* digital rectal examination, *PSA* prostate-specific antigen, *fPSA* free prostate-specific antigen, *PSAD* prostate-specific antigen density, *PV* prostate volume, *neg* negative, *pos* positive

^a^Hypoechoic masses and microcalcifications were observed using ultrasound

**Table 2:** Multivariate analysis of the predictors of prostate cancer

Variables	B	SE	OR	95 % CI	P
Intercept	2.996	0.949			0.002
PV	−0.048	0.007	0.953	0.940–0.966	<0.001
Age	0.085	0.014	1.088	1.060–1.118	<0.001
Abnormal DRE^a^	1.611	0.257	0.2	0.121–0.331	<0.001
Hypoechoic^a^	0.871	0.205	0.419	0.280–0.626	<0.001
f/t	−7.148	1.638	0.001	0–0.02	<0.001
PSA	0.094	0.024	1.099	1.048–1.152	<0.001

^a^Reference category was negative

**Table 3:** The positive prostate biopsy rate of new score system

Score	Neg	Pos	(%)
	N	N	
0	48	1	2
1	152	14	8.4
2	216	35	13.9
3	129	65	33.5
4	34	60	63.8
5	10	30	75
6	0	3	100

*Neg* negative, *Pos* positive

**Fig. 1:**
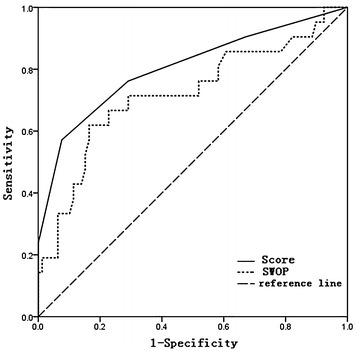
ROC of our new score system and SWOP. The AUC of these predictors were 0.804 and 0.720

Receiver operating characteristic (ROC) was used to evaluated the accuracy of PCa detection in the new score system (Fig. 1). A notable rise of the area under the curve (AUC) of the ROC for the new score system (0.804) was observed by using the Z-test in this study when compared with The Prostate Cancer Risk Calculator by SWOP (0.72, P = 0.002).

## Discussion

PSA is a conventional clinical screening parameter. However, the increase of PSA can also been observed in various situation such as benign prostatic hypertrophy (BPH), inflammation and prostate cancer (Littrup et al. 1994). Screening of PSA was also questioned by some scholars because although it increase the detection rate, the mortality is still not reduce and also bring the problem of overdiagnosis and overtreatment (Peres 2013). The number of patients with PSA level ranging from 4 to 20 ng/mL in the initial clinic examination is increasing since the PSA level was been an event of the routine examination in some medical center, China. A large amount of unnecessary prostate biopsy will be performed if we still insist on PSA > 4 as the biopsy indication (Wolf et al. 2010). Considering of the low specificity of the screening of PSA, some other upgraded predictors such as PSAD, f/T and The Prostate Cancer Risk Calculator by SWOP were introduced. However, most of these parameters have involved from PSA, therefore making them provincial (Vickers et al. 2009).

Previous research has shown that predictive models which based on clinical laboratory and ultrasound parameters, DRE findings can improve the accuracy of prostate cancer detection to a various degrees (Carlson et al. 1998; Eastham et al. 1999; Potter et al. 2001). Unfortunately, score systems based other races usually unsuitable for Chinese men. Furthermore, it is hard to use the score model in clinic because it remains controversial (Park et al. 2011). Thus, it is obvious that particularly designed for the patients with PSA level ranging from 4 to 20 ng/mL can gain further insights. In this study we develop a new score system to improve the accuracy of prostate cancer in PSA from 4 to 20 ng/mL.

10–14 cores biopsy has gained widespread acceptance in the initial examination (Scattoni et al. 2007; Ukimura et al. 2013). We used 12 + 1 cores biopsy with ultrasound and got high accuracy of PCa detection, which guarantee the dependability of our new score system.

The positive rate of patients had positive biopsy results in this study was more than 50 % when score >3, which means patients with 0 and 1 score had a low positive rate of biopsy (<10 %). Encourage patients with 0 and 1 score to receive prostate biopsy may lead to overdiagnosis and overtreatment.

ROC curve is widely used to evaluate clinic diagnosis index. AUC shows a positive correlation with the value of diagnosis index. AUC of our new score system was 0.790, which was significantly higher than The Prostate Cancer Risk Calculator by SWOP (0.720, P = 0.002) (Fig. 1). The accuracy of PCa detection of the new score system is significantly higher than single parameter.

Prostate biopsy belongs to invasive examination and has some risks including bleeding and infection. PCa is a common malignant disease in elderly people, who are also suffering from the threaten of some chronic disease such as hypertension, diabetes and coronary artery disease. Previous studies have demonstrated that the accuracy of PCa detection of patients with PSA level ranging from 4 to 20 ng/mL is low. Thus, elderly patients should undergo biopsies depended on their physical conditions. A new score system was developed based on our study. According to the system, patients with 0–1 score should closely monitored for PSA; patients with 2 score should consider to biopsy according to physical examinations; patients with score ≥3 should undergo biopsy actively. The other advantage of the new score system is that predictors such as PSA, PV, age, DRE finding, hypoechoic mass and f/T are routine clinical examination events. It is convenient to use new score system on outpatients instead of complex formulas.

## Conclusions

In conclusion, we developed a new score system to improve the accuracy of prostate cancer detection in PSA 4–20 ng/mL. A reasonable prostate biopsy strategy based on the new score system was demonstrated to reduce the unnecessary prostate biopsies and increase the detection rate of PCa.
